# Live video from bystanders’ smartphones to medical dispatchers in real emergencies

**DOI:** 10.1186/s12873-021-00493-5

**Published:** 2021-09-06

**Authors:** Gitte Linderoth, Freddy Lippert, Doris Østergaard, Annette K. Ersbøll, Christian S. Meyhoff, Fredrik Folke, Helle C. Christensen

**Affiliations:** 1 Copenhagen Emergency Medical Services, University of Copenhagen, Telegrafvej 5, DK-2750 Copenhagen, Denmark; 2 Department of Anaesthesia and Intensive Care, Copenhagen University Hospital – Bispebjerg and Frederiksberg, Copenhagen, Denmark; 3 Department of Clinical Medicine, University of Copenhagen, Copenhagen, Denmark; 4 Copenhagen Academy for Medical Education and Simulation, CAMES, Copenhagen University Hospital –Herlev, Copenhagen, Denmark; 5 National Institute for Public Health, University of Southern Denmark, Copenhagen, Denmark; 6 Department of Cardiology, Copenhagen University Hospital – Gentofte, Copenhagen, Denmark; 7 Danish Clinical Quality Program (RKKP) ▪ National Clinical Registries, Copenhagen, Denmark

**Keywords:** Emergency medical dispatcher, Telephone triage, Telemedicine, Emergency medical service, Dispatcher, Telehealth, Videoconference, Health technology

## Abstract

**Background:**

Medical dispatchers have limited information to assess the appropriate emergency response when citizens call the emergency number. We explored whether live video from bystanders’ smartphones changed emergency response and was beneficial for the dispatcher and caller.

**Methods:**

From June 2019 to February 2020, all medical dispatchers could add live video to the emergency calls at Copenhagen Emergency Medical Services, Denmark. Live video was established with a text message link sent to the caller’s smartphone using GoodSAM®. To avoid delayed emergency response if the video transmission failed, the medical dispatcher had to determine the emergency response before adding live video to the call. We conducted a cohort study with a historical reference group. Emergency response and cause of the call were registered within the dispatch system. After each video, the dispatcher and caller were given a questionnaire about their experience.

**Results:**

Adding live video succeeded in 838 emergencies (82.2% of attempted video transmissions) and follow-up was possible in 700 emergency calls. The dispatchers’ assessment of the patients’ condition changed in 51.1% of the calls (condition more critical in 12.9% and less critical in 38.2%), resulting in changed emergency response in 27.5% of the cases after receiving the video (OR 1.58, 95% CI: 1.30–1.91) compared to calls without video. Video was added more frequently in cases with sick children or unconscious patients compared with normal emergency calls. The dispatcher recognized other or different disease/trauma in 9.9% and found that patient care, such as the quality of cardiopulmonary resuscitation, obstructed airway or position of the patient, improved in 28.4% of the emergencies. Only 111 callers returned the questionnaire, 97.3% of whom felt that live video should be implemented.

**Conclusions:**

It is technically feasible to add live video to emergency calls. The medical dispatcher’s perception of the patient changed in about half of cases. The odds for changing emergency response were 58% higher when video was added to the call. However, use of live video is challenging with the existing dispatch protocols, and further implementation science is necessary.

**Supplementary Information:**

The online version contains supplementary material available at 10.1186/s12873-021-00493-5.

## Background

When citizens experience a medical emergency and call for help, they contact an emergency medical dispatcher who can guide in first aid, including cardiopulmonary resuscitation [1], at the same time, they are gatekeepers to the provision of pre-hospital resources [2]. It is important to allocate the correct resources to avoid delay in time-critical conditions [3] and to minimize over-triage, which increases demands on ambulance capacity and overcrowding of the emergency departments [4]. Several studies have addressed the difficulties in selecting the optimal ambulance dispatch [5–10]. Evaluation provided by ambulance crews at arrival at the scene can be quite different from that of the dispatchers. Of the emergencies given a high priority level by the dispatchers, the ambulance crew agreed with the priority in 27% of the cases [5], and 34% of patients who were assigned an ambulance did not need the ambulance service [6]. In interview studies, medical dispatchers have described not being able to see the patient with their own eyes as a significant obstacle [7, 11]. Telehealth with videoconferencing is a significant and fast-growing modality of care. However, the main use of videoconferencing within the clinical setting is for remote consultation [12–14], and live video in the emergency dispatch centres has only been studied sparsely [15, 16]. A small feasibility study, including 21 trauma cases from the United Kingdom, found that live video from a bystander’s phone could provide dispatchers with more information from the scene and the clinical condition of the patients [15]. Video-capable smartphones are widespread; more than 88% of adults in the Denmark own such a device [17]. The use of live video from bystander’s smartphone to the medical dispatcher could be both technical feasibly and beneficial in a 1–1-2 setting.

Our aim was to assess feasibility and dispatchers’ perceptions and response after adding live video from bystanders in emergency calls at Copenhagen Emergency Medical Services (EMS), Denmark. Our primary outcome was a change in the dispatchers’ emergency response after adding live video to the emergency call, and secondary outcomes included changed assessment of the patient by the dispatcher and whether live video was beneficial for the dispatcher and the caller.

## Methods

### Setting

The study was conducted at Copenhagen EMS, which covers a population of approximately 1.84 million people and an area of 2559 km^2^. In case of an emergency, there is a single emergency phone number (112) to a call centre that identifies the need for police, fire or medical assistance. If the problem is medical, the caller is re-directed to the EMS, where medical dispatchers answer, process, and respond to the call by activating an EMS response and delivering medical advice [2]. The EMS receive approximately 110,000 emergency calls each year [18]. The medical dispatchers are specially trained registered nurses or paramedics and their decision-making process is supported by a nationwide criteria-based Emergency Medical Dispatch System (Danish Index) [2, 19], which is a tool for managing emergency calls. Within Danish Index all emergency calls are categorized into 39 different main symptom-based criteria. The categorization leads to questions that enable the medical dispatcher to divide calls into five emergency priority levels with corresponding emergency response, ranging from immediate ambulance response with lights and sirens, to medical advice or self-transportation to the hospital. Different staff can be dispatched to correspond to the patients’ needs; for example, medical emergency physician (Mobile Critical Care Unit or Helicopter Emergency Medical Services (HEMS)), paramedic, nurse or ambulance crew. In case of cardiac arrest, volunteer citizen first responders can be activated. The Danish Index for Emergency Care is integrated in the computer-assisted dispatching system provided by Logis Solutions, Denmark (Logis CAD) [20].

### Procedure

**Fig. 1:**
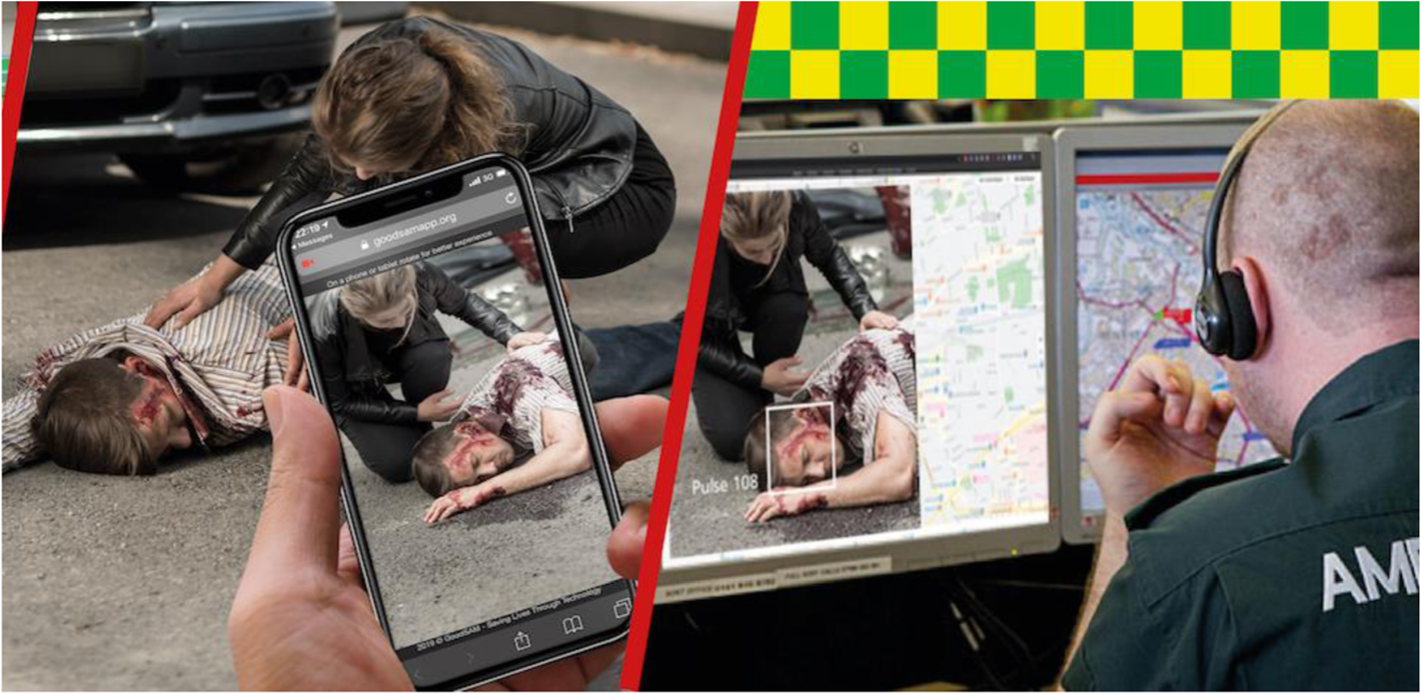
Illustration of technical solution for live video transmission from bystander’s smartphone provided by GoodSAM Instant-on-scene (www.goodsamapp.org)

**Fig. 2:**
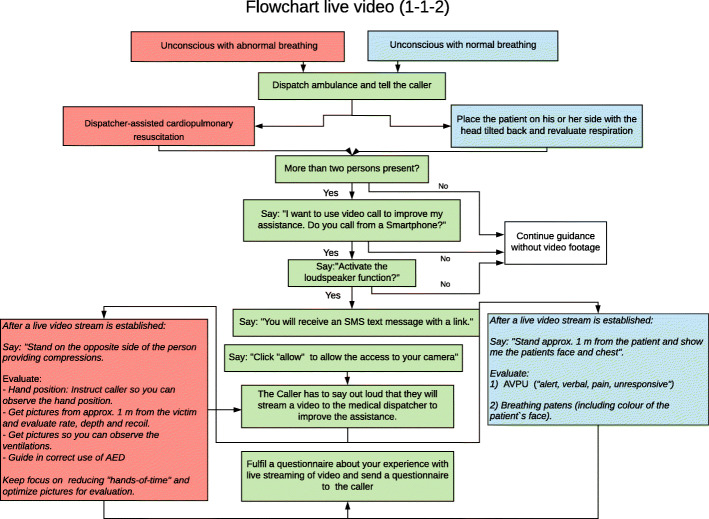
Flowchart for the medical dispatchers about adding live video from bystanders to the emergency call at Copenhagen Emergency Medical Services, Copenhagen

From June 2019 until February 2020, all medical dispatchers at Copenhagen EMS could add live video to the emergency call (see Fig. 1). We conducted a cohort study with a historical reference group. Before implementation we conducted a pilot project from October 2018 until January 2019 that included nine medical dispatchers to evaluate the feasibility of adding video to the emergency call and evaluate the questionnaire. The pilot project resulted in changes in the questionnaire regarding patient treatment, recognition of other conditions after video, and changes were also made in some formulations and outcome categories. All medical dispatchers received half a day of education in adding live video to the emergency call. The training included simulation-based scenarios and an introduction to the questionnaires. Issues such as callers prioritizing filming instead of helping the patient was included in our training of the medical dispatchers [21]. A flowchart for adding live video to the emergency call was also part of the training and available at each of the dispatcher’s workstation afterwards (Fig. 2). To avoid delayed emergency response if the video transmission failed, the medical dispatcher had to determine the appropriate emergency response before adding live video to the call. Inclusion criteria were that the caller was by the patient’s side, the estimated age of the caller was more than 18 years, a video-capable smartphone was present, and that more than two bystanders were present in the case of cardiac arrest. Each medical dispatcher could choose which emergency calls they wanted to add live video to, but they were recommended to use video if the patient was unconscious, including cardiac arrest.

### The technical solution for livestreaming of video

The technical solution was provided by GoodSAM Instant-on-scene (www.goodsamapp.org), Unite Kingdom [22]. The platform contains a technical solution designed for the pre-hospital environment. Copenhagen EMS only used location and the live video function. For livestreaming of video, the dispatcher sends a text message to the caller, asking him or her for consent to share livestream video from the camera on their smartphone. After confirmation from the bystander, the smartphone automatically started transmitting a secure video livestream from the scene to the medical dispatcher. When the emergency call was finished, the link was inactivated. During the pilot project we experienced late delivery of text messages, so we changed the delivery priority, forwarding the text messages ahead of other tele-activities.

### Data collection and outcome

Emergency response, date and time of the call, and symptom-based criteria from Danish Index were collected using the dispatch system Logis CAD. If video was used, it was registered. As a reference group, we choose all emergency calls in the time period just before the implementation of the video solution to minimized other factors that could affect the dispatch process (October 2018 to May 2019).

All changes in emergency response including allocation of staff were included in the primary analysis. Secondly, the emergency response was divided into four main responses: Emergency response with lights and siren, emergency response without lights and siren, non-urgent ambulance, or medical advice or self-transportation. Non-urgent ambulance included all non-urgent transportation provided from EMS. The last category included medical advice, referral to a general practitioner, or referral the patient to the emergency department without transportation provided by EMS. After each attempt to add live video to the emergency call, the medical dispatcher filled out an electronic questionnaire about their experience (Additional file 1). The questionnaire included whether their assessment of the patient changed after the application of live video. The medical dispatcher evaluated unconscious patients without cardiac arrest using the AVPU scale (alert, verbal, pain, unresponsive) with help from the bystanders. Other aspects were the dispatcher’s evaluation of using live video, changed treatment of the patient after video, their cooperation with the bystander, and the quality of the live video received (‘good’, ‘medium’ or ‘poor’). The value ‘medium’ was provided if the video sometimes lacked or froze, and ‘poor’ was given if the video was almost useless. Only data collected from the dispatch system was available for the reference group.

After the live video had finished, the caller received a text message containing a questionnaire about their experience (Additional file 2). The questions were conducted after telephone interviews with callers who had streamed video to the medical dispatcher in the pilot project and tested by non-medical individuals. Both questionnaires were conducted with the Electronic Data Capture system (*REDCap*), which is an open-source system developed by Vanderbilt University [23].

### Statistical analyses

Data were analysed using descriptive statistics by means of frequencies (N) and percentages (%). Associations between categorical variables were analysed with Chi-Square test. Student’s *t*-test was used to analyse the association between video use and duration of the call. Missing data was excluded from the analyse. Logistic regression analysis was used to examine the association between adding live video to the emergency call and change in emergency response. The primary analysis was adjusted for age (divided into 10-year intervals), the main criteria from Danish Index (divided into the 20 most commonly used items, other reasons and missing), and the patient’s gender. Results are reported with odds ratios (OR) and 95% confidence intervals (CI) and *p*-values where appropriate. If missing value for the confounding variable if was excluded from the analyse. Statistical significance was defined as *p* < 0.05. All analyses were performed using SAS Enterprise Guide version 7.1 statistical software.

## Results

**Fig. 3:**
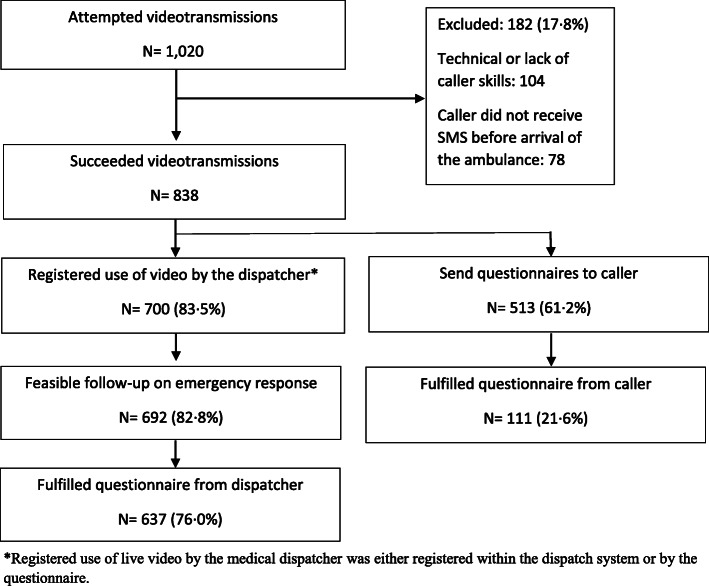
Flowchart of attempted and succeeded live video transmission from bystanders’ smartphones to the medical dispatchers, and response rates from the medical dispatchers and callers

Live video was attempted in 1.4% (1020/73,442) of all emergency calls; it succeeded in 838 (82.2%) of these calls, and follow-up was possible in 700 calls (83.5%) (Flowchart, Fig. 3). Video was used more frequently in cases with children; 26.7% were younger than 9 years of age, compared to only 5.8% in normal emergency calls (*p* < 0.001). Patients were more often unconscious and experiencing seizures compared with other emergency calls without video, where chest pain, paralysis, and ‘unclear problem’ were more frequent. The duration of the calls with video was longer (423 s vs. 242 s, p < 0.001), and less frequent at night-time (14.7% vs. 21.9%, p < 0.001), compared with the reference group without video (Table 1).

**Table 1:** Description of emergency calls where live video from bystanders’ smartphones were added compared with retrospective emergency calls without video. Data are given by frequency (N) and percentage (%)

	Live video *N* = 700	No-Video *N* = 60,216
Patient characteristics		
Male patient	314 (53.7%)	26,183 (51.6%)
Patient age (years)		
0–9	156 (26.7%)	2923 (5.7%)
10–39	193 (33.0%)	12,156 (24.0%)
40–79	189, (32.3%)	27,342 (53.9%)
≥ 80	47 (8.0%)	8313 (16.4%)
Final main Criteria/symptom^a^		
Unconscious (lifeless) adult (from puberty)	87 (12.4%)	1649 (2.8%)
Seizures / convulsions	64 (9.1%)	2576 (4.3%)
Accidents	60 (8.6%)	6225 (10.5%)
Wounds, fractures, minor injuries	51 (7.3%)	3574 (6.0%)
Intoxication, poisoning, drug overdose	48 (6.9%)	3407 (5.7%)
Sick children	44 (6.3%)	563 (1.0%)
Altered levels of consciousness / paralysis	39 (5.6%)	6159 (10.4%)
Unclear problem	38 (5.4%)	5990 (10.1%)
Breathing difficulties	32 (4.6%)	4243 (7.2%)
Allergic reaction	23 (3.3%)	492 (0.8%)
Chest pain	11 (1.6%)	6262 (10.5%)
Call characteristics		
Daytime (07:00–14:59)	281 (40.6%)	23,910 (40.2%)
Evening (15:00–22:59)	310 (44.7%)	22,544 (37.9%)
Night-time (23:00–06:59)	102 (14.7%)	12,997 (21.8%)
Length of call (sec), mean (SD)	423 (202)	242 (145)
Weekend	222 (32.0%)	19,490 (32.8%)

^a^Main criteria is the final main criteria given from Danish Index to the emergency call. Presented are the ten most frequent used criteria when adding video and last the main critera with the largest difference between the calls with video and without video

Missing values: Patients characteristics (*n* = 115 and *n* = 8780), Final main Criteria/symptom (*n* = 88 and *n* = 5546), and Call characteristics (n = 7 and *n* = 765), for emergency calls with live video and for the reference group without video, respectively

The medical dispatchers’ response rate for the questionnaire was 76.0% (637/838). The dispatchers found the video ‘extremely useful’ or ‘very useful’ in 88.6% of the emergencies and evaluated the quality of the video received as good in 69.6% of cases (*n* = 437/628), medium in 23.7% (*n* = 149/628), and poor in 6.7% (*n* = 42/628) according to the questionnaire. In three cases the audio connection with the caller ended after the live video transmission started.

### Assessment of the patient after adding live video

The medical dispatchers stated that their assessment of the patient’s condition changed in 51.1% of the calls (condition more critical in 12.9% vs. less critical in 38.2%) after live video was available, according to questionnaire (Table 2); for example, cardiac arrest was identified in four calls after video was established. The dispatchers assessed the patients to be unconscious in 264 cases, with cardiac arrest in 60 cases, according to the questionnaire. The dispatcher’s provided APVU score changed in 115 patients (43.5%) after they used the video to evaluate it, with increased level of consciousness in 74 patients and decreased level in 41 patients. The medical dispatchers’ assessment of the breathing pattern changed for the unconscious patients in 35.2% (*n* = 70/199). More breathing difficulties in 9.5% (*n* = 19/199) included obstructed airways in five cases. The dispatcher recognized other or different disease/trauma in 9.9% of the emergencies. Registered changes within the symptom-based categories in Dansk Index occurred in 14.8% of calls with video and 10.5% for the reference group without video. Dispatchers found that patient care, such as the position of the patient, quality of cardiopulmonary resuscitation or obstructed airway, improved in 28.4% (*n* = 165/580) of emergencies.

**Table 2:** Change of the medical dispatchers` situation awareness according to the questionnaire

	Yes	No	Do not know
Changed assessment of the patient? (*N* = 636)	325 (51.1%) 82 (12.9%) More critical ill 243 (38.2%) less critical ill	273 (42.9%)	38 (5.9%)
Did the patient have another disease/condition/trauma you recognized after the live video? (*N* = 605)	60 (9.9%)	420 (69.4%)	125 (20.6%)
Changed treatment of the patient before arrival of the ambulance? (*N* = 580)	165 (28.4%) Improved treatment 2 (0.3%) Impaired treatment	352 (60.7%)	61 (10.5%)
Changed situation awareness regarding the surroundings or bystanders present? (*N* = 622)	113 (18.2%) 7 (1.1%) Less bystanders present 68 (10.9%) More bystanders present 13 (2.1%) Different surroundings	509 (81.8%)	..

The questionnaire was fulfilled after they received live video from bystander’s smartphone. Results are given by frequency (N) and percentage (%). The number presented are the responders for each question

### Emergency response after adding live video

The emergency ambulance response with lights and siren was made in 56.4% of the cases using video, compared with 43.2% in the reference group (*p* < 0.001) (Table 3).

**Table 3:** Changed emergency response during the call for emergencies where video was added from bystanders’ smartphone compared to retrospective emergency calls without video

	Provided emergency response		Changed emergency response					
	Live Video *N* = 692	No-Video *N* = 59,070	Live Video *N* = 692	No-Video *N* = 59,070	Unadjusted OR (95%CI)	*P*-value	Adjusted OR (95% CI)	*P*-value
All emergency responses			190 (27.5%)	9607 (16.3%)	1.95 (1.64–2.30)	< 0.001	1.58 (1.30–1.91)	< 0.001
Emergency response with lights and siren	395 (56.4%)	25,690 (43.2%)	138 (34.9%)	5555 (21.6%)	1.95 (1.58–2.40)	< 0.001	1.60 (1.27–2.03)	< 0.001
Emergency response without lights and siren	121 (17.3%)	19,190 (32.2%)	23 (19.0%)	1529 (8.0%)	2.71 (1.7–4.29)	< 0.001	1.84 (1.07–3.15)	< 0.001
Non- urgent ambulance	15 (2.1%)	1731 (2.9%)	4 (26.7%)	381 (22.0%)	1.29 (0.41–4.07)	0.67	1.16 (0.33–4.13)	0.82.
Medical advice or self-transportation	161 (23 0%)	12,459 (20.9%)	25 (15.5%)	2142 (17.2%)	0.86 (0.58–1.36)	0.58	0.73 (0.43–1.23)	0.24

Only main categories are presented. Data are given by frequency (N) and percentage (%)

The medical dispatcher changed the emergency response in 27.5% of calls with video and 16.3% of calls without video. The unadjusted OR was 1.95 (95% CI: 1.64–2.30) for changing emergency response during the call if video was used. Odds for changing response was 58% higher when using live video when adjusted for age, criteria within Danish Index and gender, OR 1.58 (95% CI: 1.30–1.91) (Table 3).

Change within the four main emergency response was 11.3% for emergencies with live video vs. 8. 0% for the reference group with no video (*P*-value = 0.002). The emergency response was upgraded in 9.9% (*n* = 63/632) of the emergency cases and downgraded in 19.9% (*n* = 126/632) according to the questionnaire.

### Cooperation with callers and callers’ experiences with adding live video

Dispatchers found the cooperation with caller unchallenging (89.3% (*n* = 539/604)). The reasons that dispatchers gave for challenging cooperation were: the caller was emotionally distressed (23 calls, 3.8%), language barriers (14 calls, 2.3%), the caller had difficulty following instructions (12 calls, 2.0%), or the caller had barriers towards live video transmission (three cases, 0.5%). None of the other bystanders had barriers towards the video transmission.

**Fig. 4:**
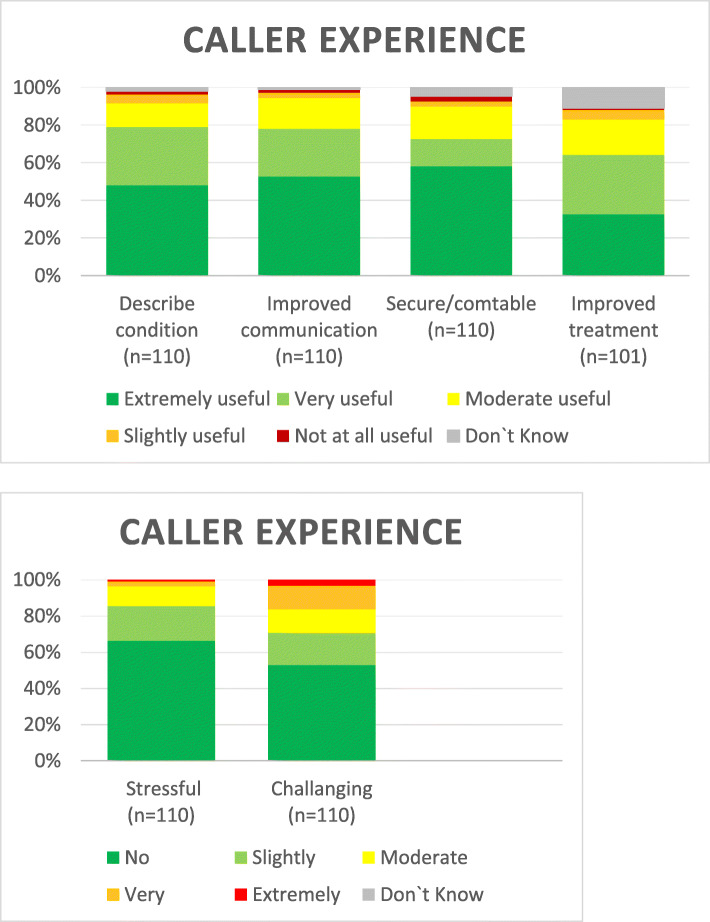
Callers experience with live video transmission to the medical dispatcher

The dispatchers sent 513 electronic questionnaires (out of 838 emergencies, 73.3%), 111 of which were received back from the callers, giving a response rate for 22% for the bystanders. General experiences were positive towards live video transmission. (Fig. 4). However, 12.5% of respondents though that it was extremely or moderately difficult to establish the video connection. Of the responders 6.5% (*n* = 7/108) were younger than 20 years and 6.5% (n = 7/108) were older than 60 years. Age was missing for three callers. The majority (97 3%) thought that the opportunity of video to EMS should be implemented, with only three saying they did not have an opinion. Ninety-nine % of callers were extremely satisfied or very satisfied with the help they received from 1 to 1-2.

## Discussion

It was feasible to add live video to the emergency call. The live video was evaluated as beneficial for the medical dispatcher, and their perceptions of the patient changed in 51.1% of the cases after receiving the video, resulting in changed emergency response in 27.5% of the emergency cases. The odds for changing emergency response were 58% higher when video was added to the call.

Our results correspond to the small feasibility study, including 21 trauma cases from UK, that used the same technical solution as in our study. Video transmission was only started if the dispatch of the HEMS was under consideration and the criteria for dispatch of the HEMS had not already been met [15]. In only five cases was the HEMS dispatched after the dispatcher had the live video from the location, and in another 14 cases the HEMS was not dispatched.

Our study found relevant changes in health outcomes, both in relation to the patient and emergency response. Communication through a video link has been studied for hospital outpatients with chronic and stable conditions [14]. Patients and staff are reported to be satisfied with the use of videoconferencing, but there is limited evidence that it led to a change in health outcomes. The emergency setting is very different involving triage and patients with an acute and potentially time critical serious condition, where evaluation of patients is essential.

We found that ‘giving eyes’ to the medical dispatcher could improve their situation awareness and thus the assistance they provide; this finding is supported by our previous study, which included closed-circuit television (CCTV) recordings of emergencies and interviews of the medical dispatchers involved. The study indicated that a medical dispatcher may not entirely understand the emergency setting from the verbal information given by caller [11]. In our study, fewer emergency calls were classified as an ‘unclear problem’ from the dispatcher compared to both normal emergency calls and what has previously been reported (17–19%) [2, 19]. Video might be preferred in cases involving patients who are difficult to assess because they are unable to talk for themselves, such as unconscious patients and small children. Video was used more frequently in these emergency cases. The dispatcher’s evaluation of the level of conscious changed in both directions; this might be partly due to time, but the time interval for video connection was rather short. The dispatchers also expressed that patient care improved in approximately one-third of cases after adding live video to the call. Simulation studies have shown that video-assisted dispatching may improve the quality of cardiopulmonary resuscitation provided compared to the audio-instructed method [24, 25]. In Seoul, South Korea, a video protocol has been implemented in case of out-of-hospital cardiac arrest [16]. The dispatcher calls the bystander back with a video call after cardiopulmonary resuscitation has started. Lee and colleagues found no difference in survival between the video-assisted group and the audio-instructed group when adjusting for potential confounders (such as age, location, witness arrest). The study did not analyse what the dispatcher saw or what was corrected on the video, and the primary call also had to be interrupted for the video call. Further exploration of the role of video in case of out-of-hospital cardiac arrest should be made.

Only a small number of emergency calls had video added. Organizational studies have shown that introducing video consultations is a complex change that disrupts long-established processes and routines [26, 27]. During the study feedback from dispatchers suggested that they did not find video necessary in many calls, as there was only a short time until arrival of the ambulance. Other explanations given by the dispatcher were changes in habit, increased workload, or to avoid a queue if many emergency calls were coming through (un-published data). Adding live video to the emergency call can potentially interfere with normal addition to the symptom-based protocol and thus delay in emergency response [20]. In our study, the dispatchers decided on the emergency response before starting the video, which also contributed to the calls having longer duration. We do not know the correct timing for adding live video to an emergency call. The patient might have been evaluated faster if the dispatcher had video from the beginning.

Although the solution seems simple, it is introduced to callers standing in the most stressful situation. Besides activating the link for video, they also must activate the loud-speaker function and accept transmission of video. In our study, 17.5% of emergency calls could not be established with a video, which is similar to that other studies have reported: 14.5% from Avest et al. [15] and 14.8% from Ecker et al. [24]. The audio calls were also disconnected in some cases, which also happened in the small feasibility study.

### Strengths and limitations

A strength of this study was that we conducted a pilot project before implementing video and trained the dispatchers with simulation before adding video to the emergency call [28]. All dispatchers had the possibility to use video and were given the survey right after the emergency call, which limited the risk of re-call bias. Response rates for dispatchers were high when considered the settings. Our outcome data from Logis CAD are of high quality because every change within Dansk Index is registered. A limitation of the study is that we cannot assess whether the changes in emergency response were appropriate because we do not have outcome data available from the ambulance crew or the hospital. We only used data from the dispatch system Logis CAD in our analyses to be able to compare with a historical reference group where video had not been used. The duration of emergency calls was longer when video was added, which itself could lead to improved situation awareness for dispatchers if more information arises during the conversation. Live video was only used in 1.4% of the calls, so a selection bias is possible since we do not know whether the dispatcher chose to add video because they had doubts about the emergency response or for another reason. Another limitation is that the study had a very low questionnaire response rate among bystanders, which means the conclusions must be considered with caution because the responses may have come from the best experiences.

### Future perspective

Further exploration is needed about how the dispatchers should approach emergency calls. Dispatchers are trained in traditional protocols based on verbal questions and answers. However, adding live video required a more comprehensive approach. More information is available, which also includes more non-relevant information increasing complexity for the decision-making process. Further exploration is also needed about cases in which video to EMS could be an advantage. Future studies could focus on potential critical patients that cannot communicate with the medical dispatcher, such as children, unconscious patients or maybe patients with languages barriers [29], which we did not focus on in this study. Implementation of new technologies can be challenging and focus on the implementation process [30] and education is necessary.

## Conclusion

It was technically feasible to add live video to the emergency call. The medical dispatchers’ perception of the patient changed in 51.1% of the cases after receiving the video, resulting in changed emergency response in 27.5% % of the emergency cases. The odds for changing emergency response were 58% higher when video was added to the call. However, implementation and use of live video are challenging with the current standard dispatch protocols and further implementation science is necessary.

## Supplementary Information

**Additional file 1.** Questionnaire to the medical dispatcher after received live video from the bystander’s smartphone.

**Additional file 2.** Questionnaire to caller after video transmission of video from their smartphone to the emergency medical dispatcher.

## Data Availability

The datasets generated and analysed during the current study are not publicly available but are available from the corresponding author on reasonable request.

## Abbreviations

EMS: Copenhagen Emergency Medical Services
HEMS: Helicopter Emergency Medical Services
Logis CAD: Computer-assisted dispatching system provided by Logis Solutions, Denmark
Danish Index: Criteria-based nationwide Emergency Medical Dispatch System, which is a tool for managing emergency calls
