# Supplementary material for: Live video from bystanders’ smartphones to medical dispatchers in real emergencies
Source: BMC Emerg Med. 2021 Sep 6;21:101. doi: 10.1186/s12873-021-00493-5 (PMC8419944; doi:10.1186/s12873-021-00493-5)
Supplement: Supplementary file 1 — Additional file 1. Questionnaire to the medical dispatcher after received live video from the bystander’s smartphone. [file 12873_2021_493_MOESM1_ESM.pdf]

Live video to the medical dispatcher

Survey text:

Please enter a questionnaire after each live video transmission attempt.

If you have any doubts about how a question should be understood, do not hesitate to contact me.

Gitte Linderoth

Email.XXX , Phonenumber XXX

(Questions with * only appear in the electronic survey if relevant from the answer above.)

(Questions regarding cardiac arrest has been removed from the survey.)

Healthcare Professional (enter the number you have been assigned	__________________
Event ID from call (copy it here):	__________________
Did the livestreaming succeed?	◯ Yes<br>◯ No
* Reason for NO video livestreaming?	◯ Bystander did not want live video<br>◯ Patient did not want live video<br>◯ Ambulance arrival<br>◯ Technical challanges
* Technical difficulties	◯ Caller does not receive SMS<br>◯ Caller cannot activate the link<br>◯ No video appeared on caller's phone<br>◯ No video appeared on your screen, but you could see the GPS marker"<br>◯ No video appeared and you could not see the GPS marker
How was the videoconnection?	◯ Good (No problems with the video connection)<br>◯ Medium (e.g. the video sometimes lacked or freezed)<br>◯ Poor (e.g. the video was lacking or froze to a degree that made it almost useless)
Reporting type ("unconscious" is when the patient is currently unable to talk to you)	◯ Unconscious with normal breathing<br>◯ Cardiac arrest<br>◯ All other causes and feedback

* Reason for the call (describe shortly)	__________________________________
* Level of consciousness : BEFORE video (AVPU)	◯ A (The patient is awake)<br>◯ V (The patient responds to verbal stimulation)<br>◯ P (The patient responds to painful stimulation)<br>◯ U (The patient is completely unresponsive)
* Level of consciousness : AFTER video (AVPU)	◯ A (The patient is awake)<br>◯ V (The patient responds to verbal stimulation)<br>◯ P (The patient responds to painful stimulation)<br>◯ U (The patient is completely unresponsive)
* Was the patient's breathing patterns different from what you thought before the video?	◯ Yes, more trouble breathing<br>◯ Yes, less trouble breathing<br>◯ No<br>◯ Do not know
* How was the breathing more troubled?	☐ Fast respiration frequency<br>☐ Obstructed airway<br>☐ Prolonged expiration<br>☐ Inspiratory stridor<br>☐ Blue skin<br>☐ Other<br>☐ Do not know
Cooperation with caller	◯ No challenges<br>◯ Challenges
* Why was the cooperation with the bystanders challenging?	☐ Bystander emotionally stressed<br>☐ Language barrier<br>☐ Caller has difficulty following all instructions<br>☐ Caller has barrier to filming<br>☐ Other people present have a barrier to being filmed<br>☐ Other
* Describe why cooperation with bystanders was difficult	__________________________________
Did your perception of the patient's condition change after the live video transmission?	◯ No<br>◯ Yes - patient is more critical ill than I thought<br>◯ Yes - patient is less critical ill than I thought<br>◯ Do not know
* How was patient sicker than you thought before the live video?	__________________________________
* How was patient less sick than you thought before the live video?	__________________________________
Did your perception of the environment or human resources change?	◯ Yes<br>◯ No

* How was your perception different? (Multiple selections are possible)	☐ More people present<br>☐ Fewer people present<br>☐ They took better care of the patient<br>☐ They took less care of the patient<br>☐ The surroundings were different<br>☐ Other
* How did they take better care of the patient?	__________________________________
* How did they take less care of the patient?	__________________________________
* How was the surrounding different?	__________________________________
* What was different than you thought before the video? (Describe briefly)	__________________________________
Was the live video useful?	◯ Extremely useful<br>◯ Very useful<br>◯ Moderate useful<br>◯ Slightly useful<br>◯ Not at all useful
Did you recognize other or different disease/trauma after the live video?	◯ Yes<br>◯ No<br>◯ Do not know
* What disease/trauma did you recognize after the video?	__________________________________
Did the livestreaming of video change the help the patient received?<br>(excluding changed emergency response)<br><br>For example, positive- stopped bleeding, better patient position. For example, negative - filming removed focus from patient treatment	◯ Yes - the patient received better help<br>◯ Yes - the patient received worse help<br>◯ No - the patient received the same help<br>◯ Do not know
* Why did the patient get better help after live video?	__________________________________
* Why did the patient get worse help after live video?	__________________________________
Did you change the ambulance response after video?	◯ No change<br>◯ Upgrade<br>◯ Downgrade
General comments and reflections	__________________________________
