# Supplementary material for: Live video from bystanders’ smartphones to medical dispatchers in real emergencies
Source: BMC Emerg Med. 2021 Sep 6;21:101. doi: 10.1186/s12873-021-00493-5 (PMC8419944; doi:10.1186/s12873-021-00493-5)
Supplement: Supplementary file 2 — Additional file 2. Questionnaire to caller after video transmission of video from their smartphone to the emergency medical dispatcher. [file 12873_2021_493_MOESM2_ESM.pdf]

Live video when calling the emergency number

Survey text:

We are trying to improve our help by supplementing the 1-1-2 conversation with video. Therefore, we would like to hear about your experience. The link is to a questionnaire with a few questions that we hope you will answer. If you do not want to participate no further action is required.

Thank you for your help.

(Questions with * only appear in the electronic survey if relevant from the answer above)

What time did you call 112?
(If you do not know the exact time, write an approximate time)

____

How difficult was it to establish the video connection?

- ◯ Extremely difficult
- ◯ Very difficult
- ◯ Moderately difficult
- ◯ Slightly difficult
- ◯ Not at all difficult
- ◯ I do not know

Was the video connection a help for you when describing the patient's condition?

- ◯ Extremely useful
- ◯ Very useful
- ◯ Moderately useful
- ◯ Slightly useful
- ◯ Not at all useful
- ◯ I do not know

Was the live video helpful when communicating with the healthcare professional?

- ◯ Extremely useful
- ◯ Very useful
- ◯ Moderately useful
- ◯ Slightly useful
- ◯ Not at all useful
- ◯ I do not know

Was it difficult to use live video?

- ◯ Extremely difficult
- ◯ Very difficult
- ◯ Moderately difficult
- ◯ Slightly difficult
- ◯ Not at all difficult
- ◯ I do not know

---

* Why was it challanging to use video?
Please decribe

____

Did the live video transmission make you feel more secure in handling the emergency?	◯ Extremely<br>◯ Very<br>◯ Moderately<br>◯ Slightly<br>◯ Not at all<br>◯ I do not know
Did it become more stressful after adding live video to the conversation?	◯ Extremely<br>◯ Very<br>◯ Moderately<br>◯ Slightly<br>◯ Not at all<br>◯ I do not know
Was the live video transmission beneficial when you were helping the patient?	◯ Extremely<br>◯ Very<br>◯ Moderately<br>◯ Slightly<br>◯ Not at all<br>◯ I do not know
Overall, are you satisfied with the help you received from the healthcare professional at 1-1-2?	◯ Extremely<br>◯ Very<br>◯ Moderately<br>◯ Slightly<br>◯ Not at all<br>◯ I do not know
Do you think live video transmission forward should be an option when calling 1-1-2?	◯ Yes<br>◯ No<br>◯ I do not know
If you have any further considerations or comments please write them here.	______________________________
What is your age?	◯ Younger than 20 years<br>◯ 20-30 years<br>◯ 31-40 years<br>◯ 41-50 years<br>◯ 51-60 years<br>◯ 61-70 years<br>◯ 71-80 years<br>◯ Older than 81 years
May we contact you if we have any follow-up questions	◯ Yes<br>◯ No
* What phone number should we use to get in contact with you?	________________________
